# Socially-Assistive Robots Using Empathy to Reduce Pain and Distress during Peripheral IV Placement in Children

**DOI:** 10.1155/2020/7935215

**Published:** 2020-04-09

**Authors:** Margaret J Trost, Grace Chrysilla, Jeffrey I. Gold, Maja Matarić

**Affiliations:** ^1^Keck School of Medicine, University of Southern California, Department of Pediatrics, Los Angeles, CA, USA; ^2^The Saban Research Institute and Children's Hospital Los Angeles, Los Angeles, CA, USA; ^3^The Boeing Company, Chicago, IL, USA; ^4^Keck School of Medicine, University of Southern California, Department of Anesthesiology, Pediatrics, and Psychiatry & Behavioral Sciences, Los Angeles, CA, USA; ^5^University of Southern California, Viterbi School of Engineering, Los Angeles, CA, USA

## Abstract

**Objectives:**

Socially-assistive robots (SAR) have been used to reduce pain and distress in children in medical settings. Patients who perceive empathic treatment have increased satisfaction and improved outcomes. We sought to determine if an empathic SAR could be developed and used to decrease pain and fear associated with peripheral IV placement in children.

**Methods:**

We conducted a pilot study of children receiving IV placement. Participating children were randomized to interact with (1) no robot, or a commercially available 3D printed humanoid SAR robot programmed with (2) empathy or (3) distraction conditions. Children and parents completed demographic surveys, and children used an adapted validated questionnaire to rate the robot's empathy on an 8-point Likert scale. Survey scores were compared by the *t*-test or chi-square test. Pain and fear were measured by self-report using the FACES and FEAR scales, and video tapes were coded using the CHEOPS and FLACC. Scores were compared using repeated measures 2-way ANOVA. This trial is registered with NCT02840942.

**Results:**

Thirty-one children with an average age of 9.6 years completed the study. For all measures, mean pain and fear scores were lowest in the empathy group immediately before and after IV placement. Children were more likely to attribute characteristics of empathy to the empathic condition (Likert score 7.24 *v*. 4.70; *p*=0.012) and to report that having the empathic vs. distraction robot made the IV hurt less (7.45 vs. 4.88; *p*=0.026).

**Conclusions:**

Children were able to identify SAR designed to display empathic characteristics and reported it helped with IV insertion pain and fear. Mean scores of self-reported or objective pain and fear scales were the lowest in the empathy group and the highest in the distraction condition before and after IV insertion. This result suggests empathy improves SAR functionality when used for painful medical procedures and informs future research into SAR for pain management.

## 1. Introduction

Painful medical procedures such as the insertion of peripheral intravenous (IV) catheters in infants and children can have long-lasting effects, such as increased sensitivity to future painful stimuli, avoidance of medical care, posttraumatic stress disorder, or even changes in neuronal architecture [1–4]. In addition to implications for patient satisfaction and safety, IV placement incurs costs related to the number of attempts and personnel time. A recent study determined that 28% of patients require 3 or more IV attempts and these patients consume 43% of total IV costs [5]. Reducing a child's perception of pain and distress associated with the procedure could lead to both increased success of placement, decreased long-term adverse effects, and reduced costs. Products currently available to reduce pain may require long application times (topical analgesics) [6], additional needle sticks (intradermal lidocaine), or are inferior to traditional methods (cold vibration) [7]; therefore, there is increased interest in new preventative techniques.

Socially-Assistive Robotics (SAR) offers a unique opportunity to mitigate pain during medical procedures. They establish communication and create a shared relationship without touching the child by utilizing embodiment, personality, empathy, and adaptation skills. These robots have been shown to reduce pain and anxiety associated with hospitalization [8, 9] and during short procedures such as vaccine administration [10] in a limited number of studies that generally use the robot solely as distraction. Preliminary work from the University of Southern California Interaction Lab has shown that SARs that display empathy create an interaction perceived as more positive by the adults [11]. In addition, empathy is recognized as a core element of the doctor-patient relationship and is associated with improved outcomes [12]. Therefore, we carried out a pilot study to test the hypothesis that empathic SAR vs. distracting SAR interactions with children reduces pain and distress in children receiving an IV in an in-hospital setting. Finally, we hypothesize that parents and children will be more satisfied and apt to want further robot interactions from the empathic SAR.

## 2. Methods

We conducted a randomized pilot study at Children's Hospital Los Angeles (CHLA), a large free-standing, academic, urban tertiary-care children's hospital from November 2015 to July 2018. A prolonged recruitment time was chosen due to study staff availability. Children arriving to the radiology suite for IV placement prior to sedated magnetic resonance imaging (MRI) and utilizing child life services were screened for inclusion. Child life specialists use play and psychological preparation as tools to help children cope with hospitalization or painful procedures, and are available in most hospitals specializing in pediatric care [13]. The MRI area of the hospital was targeted because IVs are frequently placed for sedation or contrast, and the same child life specialist helps all children in this department. We used a nonprobabilistic convenience sample to recruit children into the study. Each week, all eligible children were identified by child life using that week's MRI schedule, and all of these were approached for enrollment if study personnel were available. Inclusion criteria included children between 4 and 14 years of age who understood English (due to the SAR's English script), with English- or Spanish-speaking parents, already ordered to receive an IV by their medical team, and parent or self-report of not being afraid of robots. Children with severe developmental delay as determined by the ability to assent and parent report were excluded. All participants were offered a small honorarium ($20 gift card) for participation.

After consent, patients were randomized using a random-condition generating document (each time opened gave a new assignment) to one of the three test conditions: [1] usual child life and empathetic robot, [2] usual child life and nonempathetic robot, or [3] control, with the usual distraction services provided by child life. Randomization was performed for blocks of 6, with 2 to each condition, and was performed by a research assistant and concealed until postconsent. All study procedures were approved by the CHLA Institutional Review Board. Study recruitment occurred from 11/19/2016 to 07/08/2018. Our recruitment goal was based on a sample size that seemed feasible for this pilot study with a goal of at least 10 patients per condition. The study was terminated after 33 patients were enrolled.

After randomization, the patient's parent (or legal guardian) completed the validated the Children's Behavior Questionnaire (CBQ) to assess temperament [14], a demographic survey, the Beck Anxiety Inventory [15], and questions assessing previous experience with and pain/anxiety associated with IV placement. Children completed a baseline Medical Fears Scale (subscale of the Fear Survey Schedule) [16] and then rated their pain on a tablet computer interface using the Wong-Baker FACES scale [17] and distress using the Children's Fear Scale [18] at three time points: the beginning of the interaction, immediately prior to the IV placement, and immediately after the interaction. The tablet was also used to allow the child to select preprogrammed responses to the SAR's questions during the intervention. Following placement of the IV, parents and children in the two robot conditions also completed short surveys regarding their attitudes about the SAR. The parent survey was developed de novo for this work, while the child survey was adapted from The Young Children's Empathy Measure, which has previously been applied to social robotics research [19].

**Figure 1:**
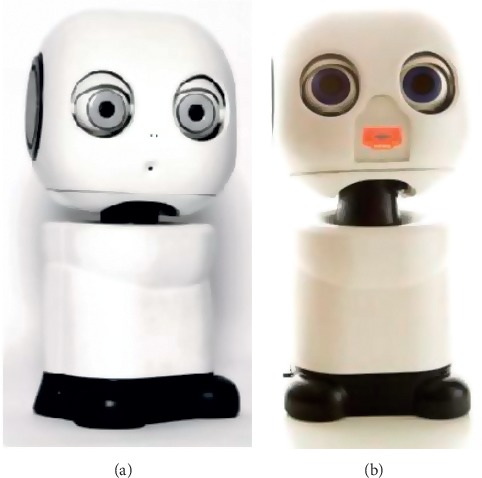
IVEY, the socially assistive robot used in this study. (a) A 3D printable robot available from Hello Robo, Inc. (b) IVEY with postproduction light-emitting diode (LED) mouth allowing simulated effect.

The SAR used for this study was the MAKI (Figure 1(a); named IVEY for this study, cost $2985), an open-source 3D printable robot designed by Hello Robo, Inc. A light-emitting diode (LED; Figure 1(b)) mouth was designed by the USC Interaction lab and added after production to allow a greater range of simulated affect by the robot. In the distraction condition, IVEY played a tablet-based dress-up game with the child. In the empathy condition, IVEY's scripted verbal responses and effect changed based on the child's expressed level of fear or pain in order to reflect the cognitive and affective components of empathy [20]. Using images on the tablet, IVEY helped the child life specialist prepare the child for IV insertion, practice deep breathing, and explain that its role was to care about the child and provide support.

**Figure 2:**
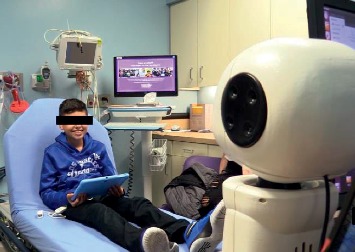
Interaction environment and setup. Patient preparing to interact with IVEY who is situated at the end of the hospital bed.

The IVEY robot interacted with children in one of the two small rooms in the radiology suite at CHLA; it stood on a small table at the foot of the child's bed and was operated by a study member (Figure 2).

All interactions were video recorded. After completion of the study, videos were reviewed by two individuals (not part of the study team, blinded to the research hypotheses) and coded for children's pain using both the Face, Legs, Activity, Cry, Consolability (FLACC), and Children's Hospital of Eastern Ontario Pain (CHEOPS) scales [21, 22]. Coders were trained and tested on a subset of videos prior to final coding (kappa = 0.832). Scores were applied at the same three time points as mentioned previously.

To evaluate success of randomization, we compared differences in demographic characteristics between the empathic SAR and comparison groups using a chi square for nominal level data (i.e., sex and age). Differences in children's pain and distress between the robot and comparison conditions were analyzed with an analysis of variance (ANOVA). Likert-scale responses to survey questions were compared between empathic and distracting robot conditions using *t*-tests. Statistical analyses were performed using *R* software [13].

## 3. Results

Thirty-three children were recruited and 31 children (average age 9.6 years) had complete data and were included in the final analysis. In general, children and their parent/guardian in all three conditions had no significant demographic differences (Table 1). One guardian in the control condition reported an extremely high level of anxiety on the Beck Anxiety scale. Children in the empathy condition had slightly lower numbers of lifetime IVs placed than in the other conditions.

### 3.1. Pain and Distress Outcomes

**Figure 3:**
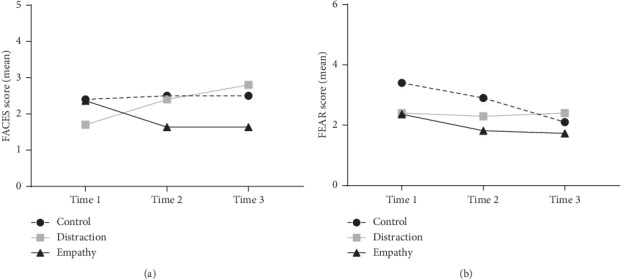
Patient-reported pain and distress scores over time. (a) Mean scores on the FACES scale showing decrease (less pain) over time in the empathy group. (b) Mean scores on the FEAR scale also showing decrease (less distress) over time in the empathy group.

Patients in the empathy arm had the lowest self-reported mean scores on the FACES scale immediately after interacting with IVEY (time point 2) and at the end of the study following IV placement (time point 3). Children in the distraction condition ended the study with the highest (worst) average scores. Two-way repeated measures ANOVA were conducted and there was no significant difference between groups (*F*(2,27) = 0.758, *p*=0.758) or over time (*F*(2,54) = 0.510, *p*=0.603) although the interaction effect was stronger (*F*(4, 54) = 2.18, *p*=0.083) due to the crossover pattern of empathy condition scores improving and control/distraction scores worsening over time (Figure 3(a)).

There was no significant difference between groups (*F*(2,27) = 0.776, *p*=0.470) on the FEAR scale, but scores did significantly reduce over time (*F*(2, 54) = 3.264, *p*=0.046). There was no significant interaction effect between group and time [*F*(4,54) = 0.968, *p*=0.433], although again the empathy group finished their interactions with the lowest scores compared to the control/distraction groups (Figure 3(b)).

**Figure 4:**
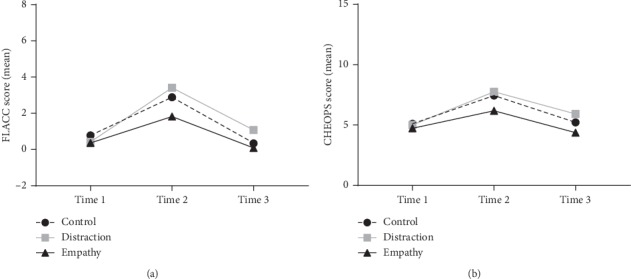
Observed pain scores over time. (a) Mean scores on the FLACC scale over time, showing increased pain at time-point 2 (IV insertion), which is lowest in the empathy group. (b) Mean scores on the CHEOPS scale over time in the same pattern.

Analysis of video recordings found similar results; video reviewers gave patients in the empathy arm the lowest mean scores on the FLACC and CHEOPS at all three time points (Figure 4). There were significant differences on two-way repeated measures ANOVA related to time with a spike in score at time-point 2 (IV placement) observed for both FLACC (*F*(2,58) = 30.06, *p* < 0.0001) and CHEOPS (*F*(2,58) = 29.66, *p* < 0.0001). However there were no significant statistical differences between groups (FLACC: *F*(2, 29) = 1.494, *p*=0.241; CHEOPS: *F*(2,29) = 2.598, *p*=0.092) or on interaction (FLACC: *F*(4, 58) = 1.261, *p*=0.296; CHEOPS: *F*(4,58) = 1.12, *p*=0.356).

### 3.2. Parent Satisfaction Outcomes

Parents of enrolled children who interacted with IVEY completed an exit survey regarding their opinions with seven items scored on a 7-point Likert scale. Overall, parents with children participating in the empathy group had more positive scores than those in the distraction group (Table 2). There was a trend towards significance with parents in the empathy condition more likely to disagree with the statement “Talking to the robot DID NOT help my child”.

### 3.3. Child Opinions About IVEY

Following interaction with either the empathy-driven or distraction version of IVEY, children were asked to complete a survey on an 8-point Likert scale. The survey was divided into subsections, three taken from the prior literature (attraction, utility, and intelligence) and two sections added for this study to determine if children [1] recognized IVEY as showing empathy and [2] if they felt IVEY helped with IV placement. Children had generally more positive responses towards the empathy condition across all subsections surveyed (Table 3). Most importantly, children interpreted that the empathy IVEY condition “has feelings” (*p*=0.11) compared to the distracting IVEY. In addition, children in the empathy condition were more likely to perceive that interacting with the empathy-condition IVEY vs. the distraction IVEY reduced the pain they felt when having an IV placed (*p*=0.026).

## 4. Discussion

This is one of the very few studies of socially assistive robots being used in real-world settings in general and the only one using empathy in an attempt to reduce pediatric pain and distress related to medical procedures. This study shows that a socially assistive robot designed to demonstrate empathy can be perceived as empathic by children interacting with it. Children said that the empathic robot helped them by talking about feelings, and that they felt less pain. Mean scores on all pain and distress scales were the lowest in the empathy group and the highest in the distraction group at the end of the study. Although not of statistical significance, this does indicate that with our robot, the empathy condition may be more clinically effective than distraction.

The consistent finding that the SAR with empathy outperformed distraction alone is critical, because simple distraction “alone” has been the long-standing standard of care for acute procedural pain management in pediatric medical centers [23]. Furthermore, the guiding principles for using books, bubbles, pinwheels, games, and videos have been seated within a framework that “distraction” was the primary mechanism of action. Previous studies have used the socially assistive robot NAO (Aldebaran Robotics®) to distract children and reduce pain during vaccination [10] and more recently during IV placement where early study results also showed no change in FACES pain scale but improvement in distress by the Observational Scale of Behavioural Distress-Revised (OSBD-R) [24]. Our study differs significantly from this work because the empathic IVEY is not acting as simple distraction. Rather, the robot uses similar techniques to those used by child life specialists to encourage better coping by the child. The empathic elements this work introduced may allow the creation of a more meaningful child-robot interaction. Another previous study specifically addressed medical-related fears and anxiety using Paro, a soft robotic seal robot. Paro acted similar to that of a companion animal and did not speak to the patient or offer any medical information [8]. The design of our study allows a more seamless integration of IVEY as a helper to the child life specialist who facilitates child coping and provides medical information.

Limitations of this study include the single-center design, restriction to English-speaking children, and convenience sampling, all of which may lead to selection bias. We attempted to control for some of these issues by expanding our participant pool to include children with Spanish-speaking parents and used prospective screening for potentially eligible patients to maximize recruitment. One of the scales used to detect pain was the FACES scale, which has been shown in some studies to be a nonaccurate pain proxy [25]. To enhance our assessment of pain, we did include observed scales applied to videotaped encounters. However, although video data were reviewed by individuals blinded to the study design, they may have been able to identify study groups based on audio or repeated behaviors. Finally, analysis of our randomized groups revealed that children in the empathy condition had a lower portion of children who had received a great many (>15) IVs in their life, but a similar proportion in each group had received no prior IV.

Importantly, our study has a small sample size. However, given the dearth of research in this challenging area, the trends in our data could inform and influence future studies. For example, our distraction-condition IVEY resulted in higher child-reported pain and fear scores at the end of the intervention than control. For this reason, we feel that continued use of the distraction IVEY is unwarranted and would focus on improving and refining the empathic IVEY for any subsequent studies.

Future directions of this research will attempt to determine the mechanisms leading to higher child satisfaction with the empathic IVEY. Biochemical, electroencephalographic, and neuroimaging data taken during child interaction with IVEY may better explain the results we report here and allow better assessment of both distress and pain. We also plan to explore the use of SAR during other painful or anxiety-provoking procedures in a hospital setting. Long-term uses of SAR could include transition between the hospital and home environment for chronically ill patients to both decrease stress of hospitalization and act as physician extenders for medical care at home.

## Figures and Tables

**Table 1:** Demographics.

Characteristic	Control (*n* = 10)	Empathy (*n* = 11)	Distraction (*n* = 10)	*p* value
*Child*				
Age (years)	9.44 (1.51)	10.00 (1.79)	9.60 (1.5)	.810
Ethnicity^*∗*^				.208
Hispanic	5 (50)	8 (73)	4 (40)	
Other	5 (50)	3 (27)	6 (60)	
Opinion re: robots^1^	3.22 (.97)	3.80 (.42)	3.63 (.52)	.190
Baseline pain^2^	1.89 (.60)	2.40 (.84)	2.10 (.88)	.378
Baseline anxiety^3^	2.78 (.83)	2.73 (1.01)	2.70 (.95)	.984
Previous pain^4^	2.36 (.74)	2.33 (7.1)	2.14 (.90)	.831
Previous anxiety^5^	1.63 (.52)	2.10 (.32)	2.10 (.88)	.209
Child lifetime IVs				.029
None	1 (10)	2 (20)	3 (30)	
1–15	3 (30)	9 (80)	3 (30)	
>15	6 (60)	0 (00)	4 (40)	
Medical fears score	7.38 (.362)	7.73 (4.58)	7.20 (2.39)	.946
*Guardian*				
Gender^*∗*^				1.00
Mother	9 (90)	10 (91)	9 (90)	
Father	1 (10)	1 (9)	1 (10)	
Age, years	36 (8.49)	35.45 (5.87)	37.30 (7.76)	.842
Beck anxiety	7.89 (15.00)	2.73 (4.15)	1.11 (.33)	.241
Education				.100
High school or less	2 (20)	7 (64)	4 (40)	
More than high school	8 (80)	4 (36)	6 (60)	

Values are mean (standard deviation) unless marked with ^*∗*^which are absolute value (%). ^1^Opinion re: robots = 5-point Likert scale, 5 = “loves them”; all other Likert Scales are 4-point scales. ^2^4 = “Always”. ^3^4 = “Never”. ^4^4 = “Terrible amount of pain”. ^5^4 = “Not anxious at all”.

**Table 2:** Parent satisfaction questions for robot conditions.

Parent question	Empathy (*n* = 11)	Distraction (*n* = 10)	*p* value
I would recommend the robot to other parents when their children get IVs	1.30 (.95)	1.27 (.47)	.936
I DO NOT want the robot the next time my child gets an IV^*∗*^	6.80 (.63)	6.00 (1.84)	.200
Having the robot made my child getting the IV easier	1.40 (1.26)	1.73 (1.10)	.537
The robot made my child have less pain	1.30 (.95)	2.00 (1.26)	.166
The robot made my child have less anxiety	1.30 (.95)	1.82 (.98)	.234
The robot seemed to understand how my child feels	1.40 (.84)	2.45 (1.57)	.071
Talking to the robot DID NOT help my child^*∗*^	6.70 (.95)	5.09 (2.34)	.056

1 = strongly agree, 7 = strongly disagree, 7-point Likert scale. ^*∗*^Negatively worded question. Mean (standard deviation) compared by the *t*-test.

**Table 3:** Child impressions of IVEY.

Characteristic	Empathy (*n* = 11)	Distraction (*n* = 10)	*p* value
^*∗*^ *Attraction*			
Good	7.64 (.81)	7.00 (1.63)	0.286
Loving	7.82 (.40)	7.00 (1.49)	0.123
Friendly (*n* = 20)	7.80 (.63)	6.90 (1.85)	0.174
Cuddly	6.27 (2.15)	4.70 (2.75)	0.165
Warm	7.45 (1.04)	5.30 (2.75)	0.039
Nice	7.64 (.92)	7.30 (1.25)	0.497
Close	6.55 (2.16)	5.00 (2.58)	0.157
TOTAL	**7.40 (.69)**	**6.17 (1.44)**	**0.030**
^*∗*^ *Utility*			
Useful	7.18 (1.40)	7.11 (1.05)	0.899
Important	7.55 (1.07)	5.78 (2.68)	0.092
Helpful	7.73 (.65)	7.33 (.87)	0.276
TOTAL	**7.48 (.92)**	**6.74 (1.09)**	**0.124**
^*∗*^ *Intelligence*			
Intelligent	7.82 (.40)	7.00 (1.12)	0.064
*Empathy*			
The robot understands how I feel	7.18 (1.08)	5.33 (2.78)	0.090
The robot has feelings	7.27 (1.27)	4.33 (2.65)	0.011
Talking about my feelings with the robot helped me	7.27 (1.10)	4.44 (2.96)	0.022
TOTAL	**7.24 (.87)**	**4.70 (2.35)**	**0.012**
*IV*			
Would recommend that my friends use the robot when they get an IV	7.45 (1.29)	7.50 (.76)	0.925
I want the robot with me the next time I get an IV	7.73 (.47)	6.50 (2.14)	0.152
Having the robot with me made getting the IV easier	7.82 (.40)	6.88 (1.36)	0.093
Having the robot with me made getting the IV hurt less	7.45 (.82)	4.88 (2.59)	0.026
TOTAL	**7.61 (.57)**	**6.44 (1.16)**	**0.025**

^*∗*^From The Young Children's Empathy Measure (see methods). 8-point Likert scale, 1 = IVEY is the opposite of this characteristic, 8 = IVEY has this characteristic. Mean (standard deviation) compared by the *t*-test.

## Data Availability

The data used to support the findings of this study are available from the corresponding author upon request.
